# Case Report of Small Cell Carcinoma of the Ovary, Hypercalcemic Type (Ovarian Rhabdoid Tumor) with *SMARCB1* Mutation: A Literature Review of a Rare and Aggressive Condition

**DOI:** 10.3390/curroncol29020037

**Published:** 2022-01-18

**Authors:** Maria Fernanda Evangelista Simões, Alexandre André Balieiro Anastácio da Costa, Tullio Novaes Silva, Lizieux Fernandes, Graziele Bovolim, Giovana Tardin Torrezan, Dirce Maria Carraro, Glauco Baiocchi, Ademir Narcizo Oliveira Menezes, Elizabeth Santana Dos Santos, Louise De Brot

**Affiliations:** AC Camargo Cancer Center, São Paulo 01000-000, Brazil; alexandreandredacosta@gmail.com (A.A.B.A.d.C.); tullionovaes@yahoo.com.br (T.N.S.); lizieux@hotmail.com (L.F.); grazibov@hotmail.com (G.B.); giovana.torrezan@accamargo.org.br (G.T.T.); dirce.carraro@accamargo.org.br (D.M.C.); glauco.baiocchi@accamargo.org.br (G.B.); ademir.menezes@accamargo.org.br (A.N.O.M.); elizabeth.santanadossantos@gmail.com (E.S.D.S.); louise.andrade@accamargo.org.br (L.D.B.)

**Keywords:** small cell carcinoma of the ovary, hypercalcemic type, ovarian cancer, *SMARCB1* mutation

## Abstract

Small cell carcinoma of the ovary, hypercalcemic type (SCCOHT) is a rare and aggressive condition that is associated with the *SMARCA4* mutation and has a dismal prognosis. It is generally diagnosed in young women. Here, we report a case of a young woman with SCCOHT harboring a rare molecular finding with a highly aggressive biological behavior. The patient had a somatic *SMARCB1* mutation instead of an expected *SMARCA4* alteration. Even though the patient was treated with high-dose chemotherapy followed by stem cell transplantation, she evolved with disease progression and died 11 months after her first symptoms appeared. We present a literature review of this rare disease and discuss the findings in the present patient in comparison to expected molecular alterations and options for SCCOHT treatment.

## 1. Introduction

Small cell carcinoma of the ovary, hypercalcemic type (SCCOHT) is a rare and aggressive form of ovarian cancer. There is little information about this cancer, with fewer than 500 cases reported in medical literature to date [1]. SCCOHT represents less than 0.01% of all ovarian malignancies [2] and is characterized by *SMARCA4* mutations in more than 95% of cases [1]. Here, we report a case of a young woman diagnosed with SCCOHT with the *SMARCB1* somatic mutation who had a dismal outcome.

## 2. Case Report

**Figure 1:**
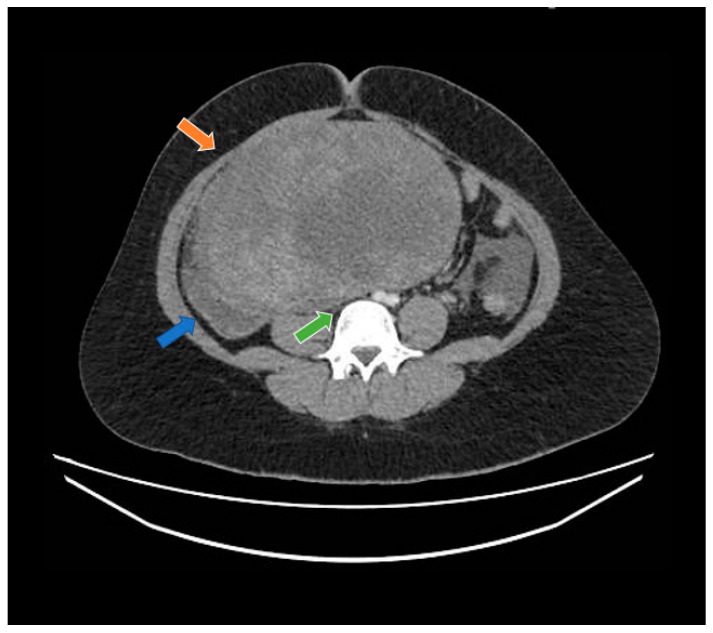
Computed tomography study in the portal phase showing a heterogeneous mass in the pelvic region (orange arrow), displacement of the intestinal loops to the right (blue arrow), and compression over the inferior vena cava (green arrow).

**Figure 2:**
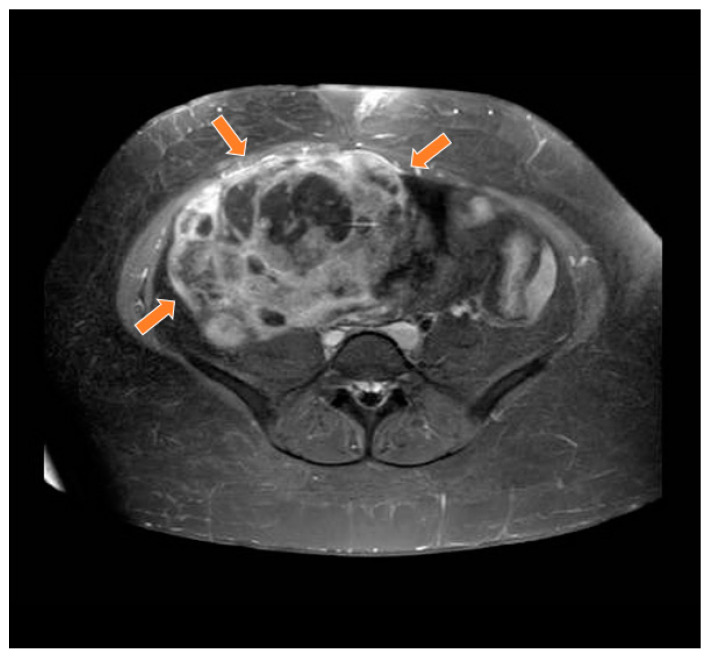
Magnetic resonance study in T1 weighted sequence post gadolinium showing a heterogeneous mass in the pelvic region (orange arrows).

A 19-year-old woman with neither comorbidities, previous pregnancy history, nor a personal or family history of cancer was referred to our healthcare service in February 2020 due to abdominal pain and swelling that had started two weeks earlier. Before starting her diagnostic investigation, the patient signed an agreement allowing the use of her clinical, radiological, and pathology information for clinical research; after that, the clinical investigation began. A physical exam uncovered a mass in the right iliac fossa topography. An abdominal computed tomography scan and magnetic resonance imaging showed a large abdominopelvic mass located on the flank and right iliac fossa, measuring 228 mm × 188 mm × 143 mm (CC × LL × AP)—Figure 1 and Figure 2, respectively. This mass occupied the mesogastrium and compressed the internal iliac vessels and the psoas, with no clear separation plane among them. The images also showed moderate ascites and retroperitoneal lymph nodes forming clusters. Initial laboratory tests showed a normal value of ionic calcium (1.3 mmol/L).

The patient underwent a mass biopsy guided by an abdominal ultrasound. A Formalin-Fixed Paraffin-Embedded (FFPE) tissue specimen demonstrated the presence of neoplasia with a biphasic pattern which was partly spindle shaped and partly epithelioid. An immunohistochemical study of the material was carried out in order to define the immunophenotype of the neoplastic cells. There was no expression of markers found in the most frequent ovarian neoplasms, such as PAX-8, p53, WT-1, inhibin, and estrogen and progesterone receptors. Other markers were present in the tissue specimen, such as cytokeratin AE1/AE3 expression, and cytokeratin staining was positive.

Focal positivity of calretinin was also observed, favoring the hypothesis of epithelial neoplasia of gynecological origin.

The patient developed worsening abdominal pain and then underwent an exploratory laparotomy in March 2020 to continue the investigation. In the intraoperative setting, a bulky mass in the left ovary measuring 300 mm was found invading the large vessels. A bulky lymph node enlargement was also found near the mass. Resections of the bulky mass, the retroperitoneal lymph node formation, and the peritoneal implants were performed, along with a salpingectomy and an oophorectomy.

The surgical specimen was composed of an ovary measuring 245 × 160 mm with a hardened and multinodular aspect. It presented a heterogeneous appearance with cystic areas that were soft and solid. Upon microscopic examination, follicle-like spaces with eosinophilic secretions were seen. Cells exhibited monomorphic round, ovoid, or spindled nuclei with vesicular chromatin, small nucleoli, and scant cytoplasm, along with rare cells showing a pattern resembling the rhabdoid morphology, as seen below in some areas with a more abundant cytoplasm, and an eccentric nucleus. Areas containing large cells were also present.

**Figure 3:**
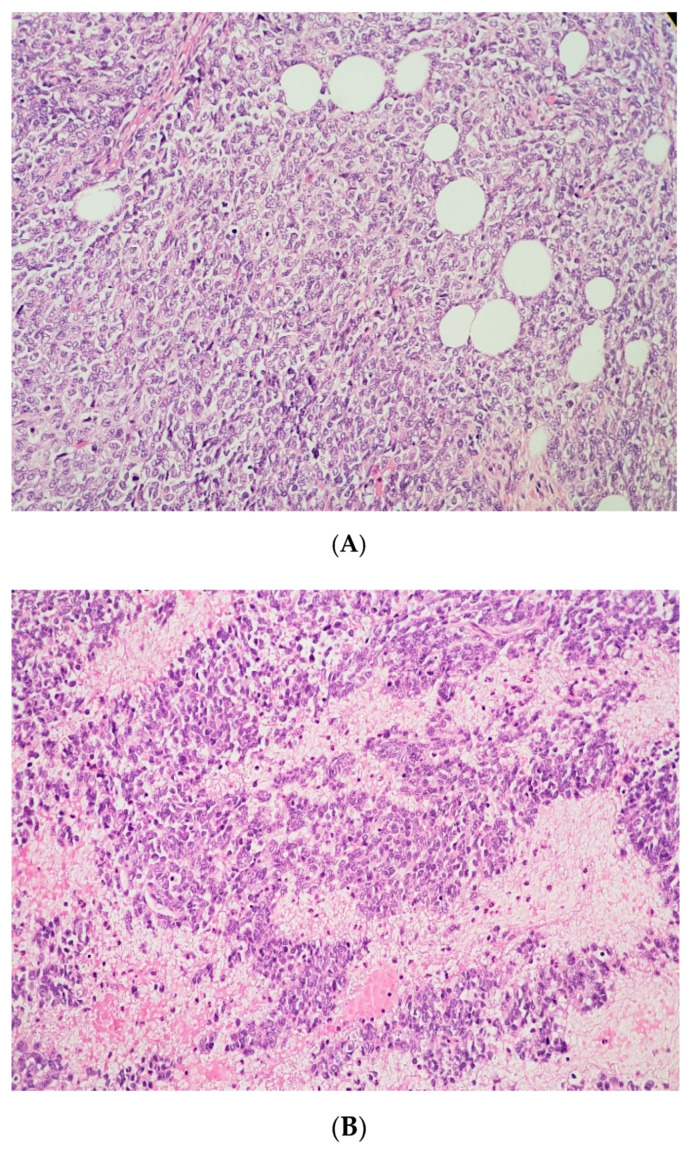

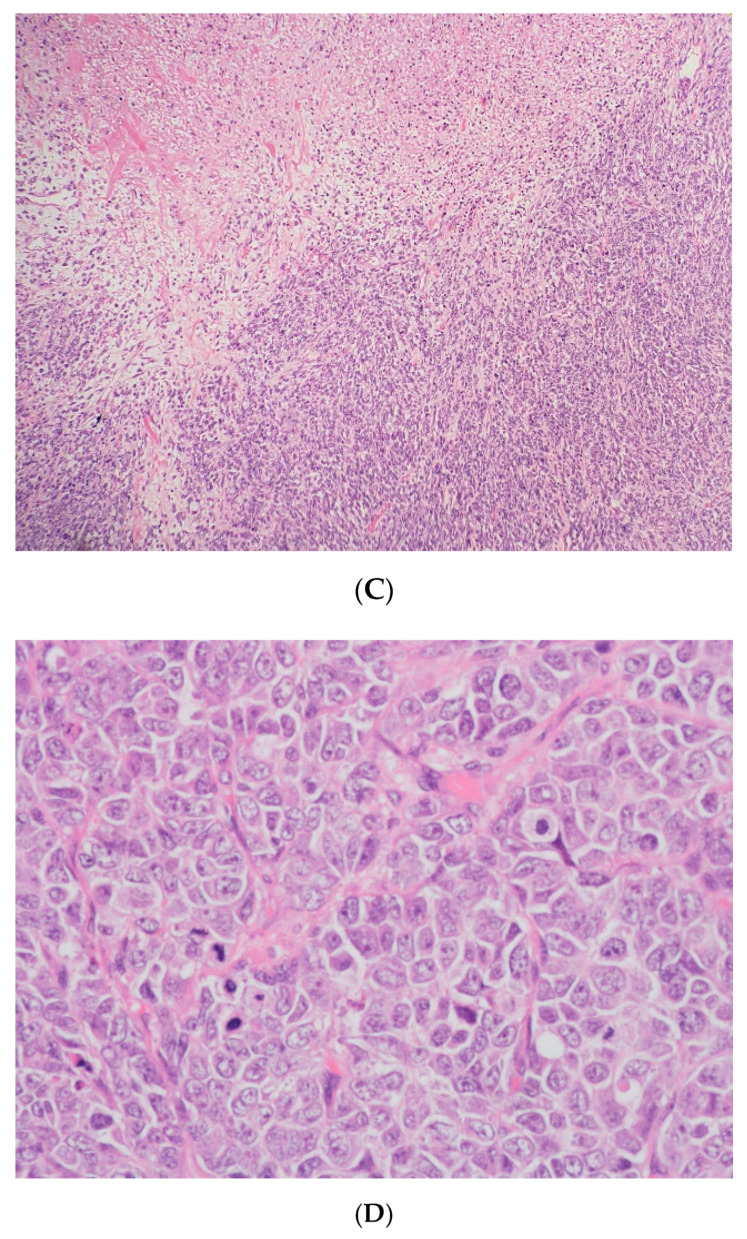
(**A**) The tumor cells are predominantly monomorphic and loosely cohesive with scant cytoplasm. (**B**) Small nests and individual tumor cells are separated by a fibrotic stroma. (**C**) A small cell component with nested growth and focal spindle morphology is juxtaposed to large cells with abundant eosinophilic cytoplasm. (**D**) The presence of areas of a more rhabdoid pattern are seen.

**Figure 4:**
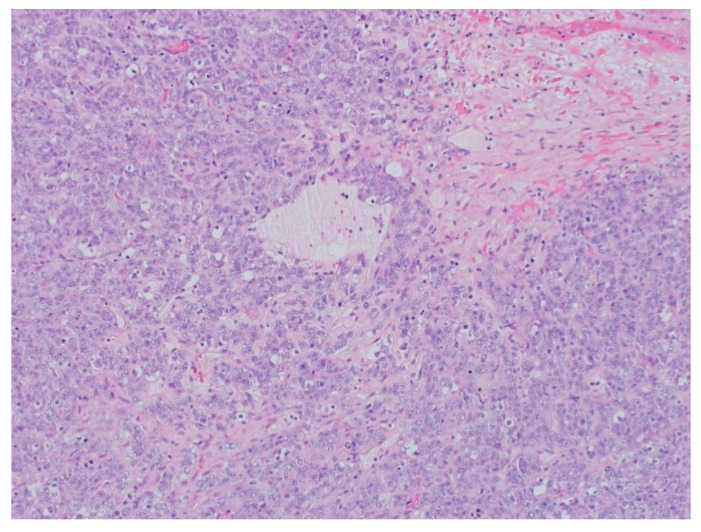
The pathology analysis of the surgical specimen, 100×. Sheets of tumor cells are interrupted by follicle-like spaces (H&E, 100×).

**Figure 5:**
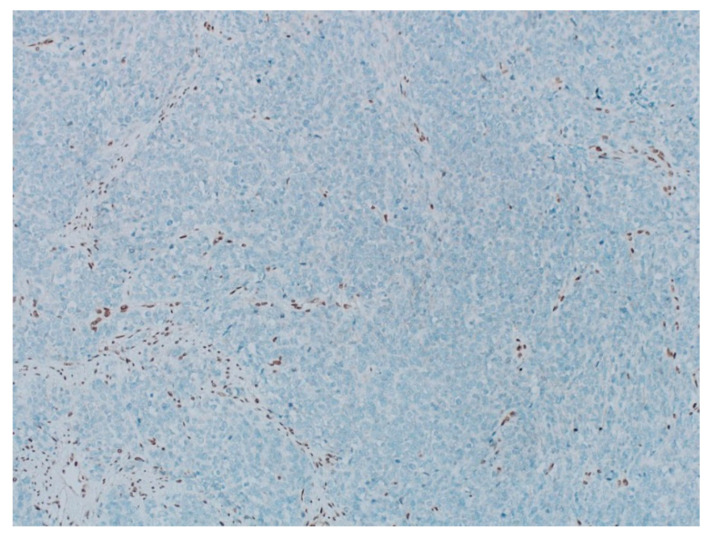
The pathology analysis of the surgical specimen. The stain INI-1—loss of expression (IHC, 100×).

**Figure 6:**
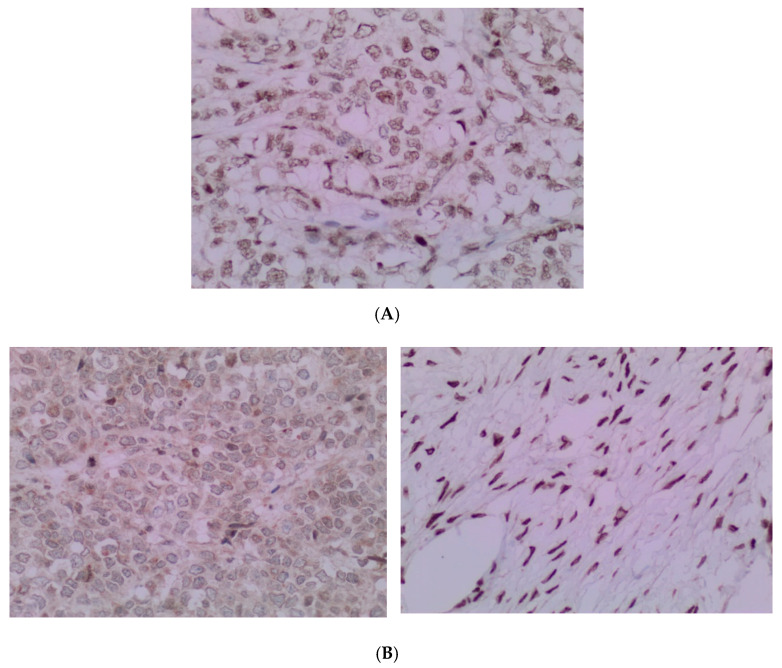
(**A**) The pathology analysis of the surgical specimen. The *SMARCA4* expression is retained (IHC, 100×). (**B**) The pathology analysis of the surgical specimen. Lack of nuclear expression of *SMARCA2* is noted (**left**) with the internal control of the *SMARCA2* in the ovarian stroma (**right**). (IHC, 100×).

The pathology analysis is presented in Figure 3, Figure 4 and Figure 5 and the immunohistochemical study showed loss of expression of INI-1 (*SMARCB1*). However, it showed the retained expression of *SMARCA4* and lack of nuclear expression of *SMARCA2* (Figure 6). The amino terminal WT-1 and EMA were negative. Neuroendocrine, epithelial, stromal, and germinal markers were also negative.

With these results, it was possible to exclude the diagnosis of the most common types of ovarian tumors, such as tumors of the ovarian surface epithelium and sex cords, as well as germ cell tumors. Based on these findings, the diagnosis of small cell carcinoma of the ovary, hypercalcemic type (SCCOHT) was suggested.

After a discussion with our molecular tumor board, some tests were proposed for the patient, such as germline genetic testing, tumor genomic testing, and personalized liquid biopsy analysis of circulating tumor DNA, or ctDNA (details of performed tests are described in Supplementary File S1). First, germline genetic testing was performed for 112 cancer-predisposing genes using a customized panel that included the *SMARCA4* and *SMARCB1* genes. No pathogenic/likely pathogenic variants or variants of uncertain significance were identified, including both point mutations and copy number variations. Then we performed paired tumor-normal Next Generation Sequencing (NGS) of a 409-gene panel using the commercial kit AmpliSeq Comprehensive Cancer Panel (Thermo Fisher Scientific, Waltham, MA, USA). Only two somatic mutations were identified in the tumor DNA: a loss of function in the *SMARCB1* gene, c.769C > T; p.(Gln257Ter), with a variant allelic frequency (VAF) of 96.3%, and a missense-damaging mutation in *PTEN* (c.510T > G; p.(Ser170Arg), VAF = 93.6%). The tumor mutation burden (TMB) estimated for this sample was less than 2 mutation per Mb. The identification of the *SMARCB1* mutation confirmed the diagnostic hypothesis of SCCOHT and the high VAF of both somatic mutations indicated loss of heterozygosity of the wild-type alleles. The ctDNA analysis was also performed and both mutations were identified in plasma (*SMARCB1* VAF = 7.2% and PTEN VAF = 4.9%). The diagnosis of SCCOHT over dedifferentiated carcinoma of the ovary was made considering not only the biological perspectives (the absence of a well differentiated component and follicle-like structures in the tumor, plus a low tumor mutational burden), but also the clinical features, especially the patient’s very young age.

Staging imaging exams showed involvement of lung and lymph node enlargement located in the level IV lymph nodes from the left cervical chain. The treatment proposed was the PAVEP protocol—Cisplatin 80 mg/m^2^ day 1, Doxorubicin 40 mg/m^2^ day 1, Etoposide 75 mg/m^2^ days 1–3, and Cyclophosphamide 300 mg/m^2^ days 1–3, every 3 weeks, and the granulocyte colony-stimulating factor on days 7–12, followed by stem cell transplantation. The patient started chemotherapy in May 2020.

**Figure 7:**
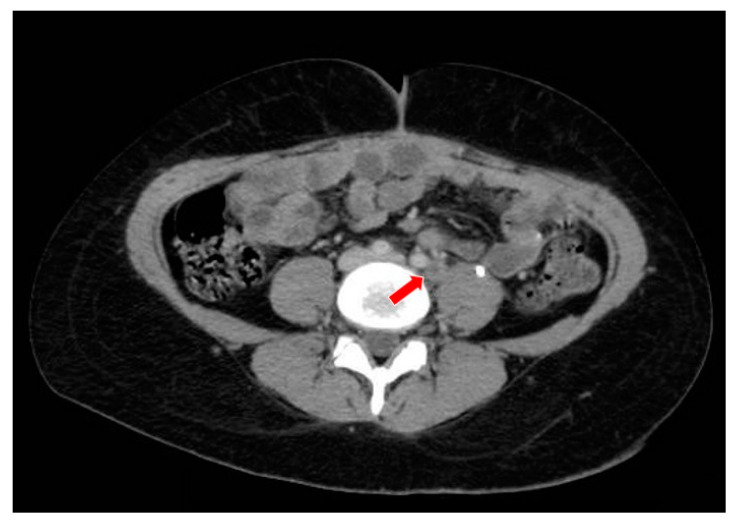
The computed tomography study in the portal phase showing a lymph node in the left iliac chain with a rounded aspect and a necrotic center (red arrow), suggestive of secondary neoplastic involvement (relapse).

After the first cycle of treatment, computed tomography showed a partial response to the PAVEP. The patient was hospitalized for febrile neutropenia after the first, second, and the fifth cycles of chemotherapy. Discontinuation of the sixth cycle of chemotherapy was decided due to medullar toxicity, and the patient proceeded to autologous stem cell transplantation in September 2020. Soon after the stem cell transplantation, disease progression was detected in the iliac and retroperitoneal lymph nodes and the peritoneum (Figure 7). The patient started second-line treatment with carboplatin and paclitaxel in November 2020.

After the first cycle of second line chemotherapy, the patient developed acute kidney injury due to extrinsic tumor compression of the ureters and a ureteral catheter was implanted. New images showed new disease progression in the retroperitoneal topography.

**Figure 8:**
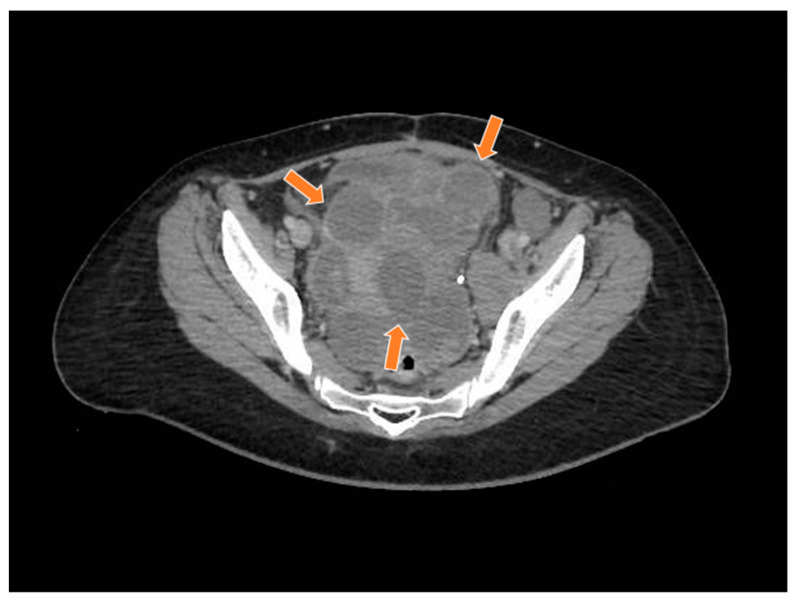
The computed tomography study in the portal phase showing a heterogeneous lesion in the pelvic region with cystic areas in between (orange arrows).

**Figure 9:**
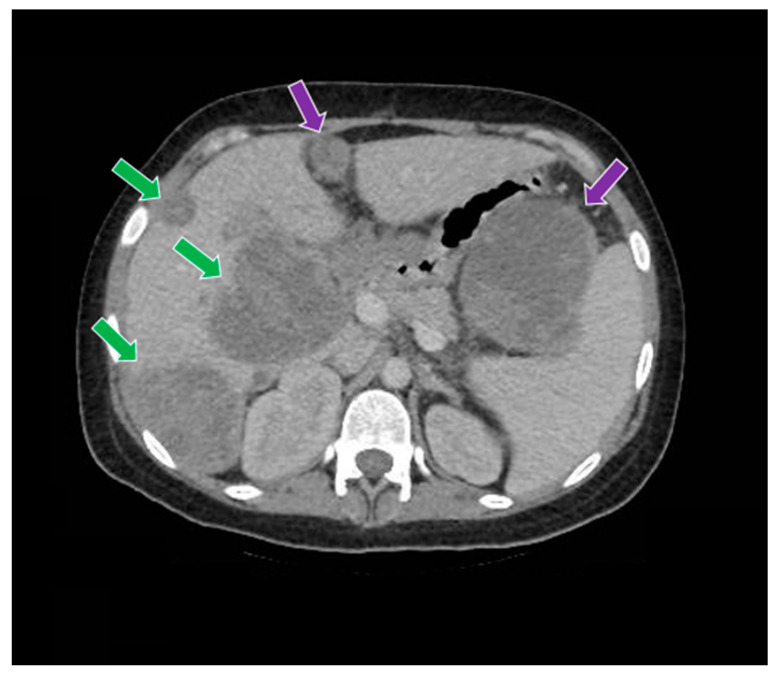
The computed tomography study in the portal phase demonstrates liver metastases (green arrows) and abdominal metastases (purple arrows).

**Figure 10:**
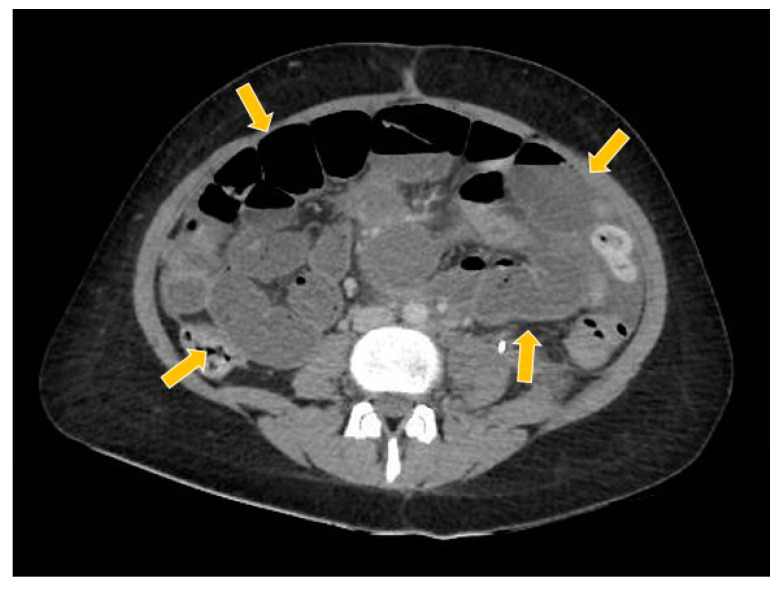
The computed tomography study in the portal phase showing the dilation of slender loops (yellow arrows) by distal extrinsic compression (new pelvic lesion).

In December 2020, a hospitalization was necessary to treat the patient’s clinical condition and to control abdominal pain. New images showed an increase in the pelvic mass, peritoneal implants, persistence of bilateral hydronephrosis, and an increase in liver nodules (Figure 8 and Figure 9). The patient was diagnosed with an intestinal sub-occlusion due to neoplastic implants in the appendix, cecum, sigmoid, and rectum (Figure 10). The patient died in January 2021.

## 3. Discussion

SCCOHT is a rare and aggressive form of ovarian cancer. The mean age at diagnosis is 23 years, with cases ranging from 9 to 43 years [3]. Even with early-stage diagnosis, the SCCOHT outcome is usually poor. The majority of patients with SCCOHT relapse and die within 2 years of diagnosis, with a long-term survival rate of only 33% [4,5].

The typical clinical presentation is a patient that has a pelvic mass that can cause pain, with 60% of women developing hypercalcemia. The mechanism underlying the commonly observed serum hypercalcemia is still not well established. A clinical study published in 1994 found that the immunoreactivity for Parathyroid hormone-related protein (PTHrP) was present in five of seven cases of ovarian small cell carcinoma [6]. Some clinical and laboratory features are associated with the prognosis. Tumor stage seems to be the most important prognostic factor. Other characteristics associated with better outcomes are patient age (<40 years), normal pre-operative calcium levels, tumor size (<10 cm), and the absence of large cells in early-stage disease [7,8].

Macroscopically, SCCOHT presents as a large-volume tumor that is predominantly solid with hemorrhagic and cystic areas. Microscopy shows diffuse growth and areas of follicular architecture, outlining nests, and trabeculae. In 80% of these tumors, variably sized follicles appeared that were empty or contained eosinophilic fluid (less often basophilic), but were often very focal. The cells have round, monotonous, hyperchromatic nuclei with small nucleoli and scant cytoplasm. Other observations include spindle cell components, large cells with broad eosinophilic cytoplasm, foci of glandular differentiation, and mucinous cells. The mitotic activity is variable, and necrosis is frequent [9]. Because of the morphologic similarity to sex cord stromal tumors and germ cell tumors, the establishment of a correct diagnosis can be challenging [10]. There are some differential diagnoses, such as granulosa cell tumor, dysgerminoma, pulmonary ovarian cell carcinoma, lymphomas, or mesenchymal neoplasms with a small-blue-round-cell pattern.

The immunohistochemistry of SCCOHT reveals positivity for cytokeratin, EMA, calretinin, and in some cases, Parathyroid Hormone (PTH). Most of the SCCOHT exhibit diffuse nuclear positivity with an antibody against the N-terminal of WT170. Desmin, S100, and inhibin are consistently negative [11]. Regarding the histogenesis of this tumor, some articles reported the hypothesis of a germ cell origin and the possible association with minute foci of a germ cell tumor [12,13].

The genomic and molecular causes of SCCOHT were initially described by multiple groups in four seminal papers [4,13,14,15]. In these publications, the pivotal role of *SMARCA4* alterations was uncovered and the pathogenic mutations in *SMARCA4*, leading to its biallelic inactivation, were shown to be the driver event for almost all cases (>90%) of SCCOHT [4,14,15]. Both somatic and germline variants in *SMARCA4* were described, with germline variants occurring in 8% to almost 40% of cases, depending on the cohort [4,14,15]. Later, a comprehensive genomic profiling of SCCOHT revealed that these tumors have a strikingly low number of somatic mutations and genomic stability, supporting the hypothesis that these tumors are largely driven by epigenetic deregulation [7,16].

It was also demonstrated that loss of the *SMARCA4* leads to loss of another SWI/SNF component, the *SMARCA2*, without underlying mutations in this last gene [17]. *SMARCA4*, *SMARCA2*, and *SMARCB1* are members of the mammalian SWI/SNF family of chromatin regulators, and about 20% of all human malignancies are associated with somatic mutations in the SWI/SNF complex [18]. It has been hypothesized that the dual deficiency in key members of the SWI/SNF complex (e.g., *SMARCA4* and *SMARCA2*, or *SMARCB1* and *SMARCA2*) can induce dedifferentiation from a normal cell or a low-grade tumor into an aggressive high-grade tumor with small cell and/or rhabdoid features [17]. Dual loss of both the *SMARCA4* and the *SMARCA2* occurs in SCCOHT and in other neoplasms such as thoracic sarcomas, undifferentiated and dedifferentiated endometrial carcinomas, and rare undifferentiated uterine sarcomas [17].

The evaluation of the *SMARCA4* alterations is performed by immunohistochemistry and genomic profiling. An article published in 2017 showed a comprehensive genomic profiling (CGP) to identify clinical and genomic alterations of SCCOHT. The CGP demonstrated the inactivating of *SMARCA4* alterations and a low tumor mutational burden (TMB) in 94% of SCCOHT cases [7]. Another clinical trial published in 2020 assessed a molecular analysis of SCCOHT, showing the presence of remarkable genomic stability with diploid profiles and low TMB. In that study, the *SMARCA4* deleterious mutations were recurrent and accompanied by the loss of expression of the *SMARCA2* [16].

According to the International SCCOHT Consortium (ISC) Guidelines, it is recommended to offer testing for the *SMARCA4* germline pathogenic variants to all patients diagnosed with SCCOHT and to refer them to a clinical genetics service. If there is no *SMARCA4* mutation found, ISC recommends that a SCCOHT diagnosis should be reconsidered [1] and should be performed by the sequencing of other related genes, such as a *SMARCB1*. In our case report, instead of a loss-of-function *SMARCA4* alteration, which is classically found in these tumors, the NGS found two somatic mutations: a loss of function in the *SMARCB1* gene and a missense-damaging mutation in the *PTEN*. This finding supports the hypothesis that the *SMARCB1* inactivation can also promote the development of SCCOHT [19].

Mutations of the *SMARCB1* gene were described in other tumors, such as epithelioid sarcomas, and in myoepithelial carcinomas [11], and most importantly, in rhabdoid tumors (RTs) [1]. In RTs, a *SMARCB1* biallelic inactivation is detected in >95% of cases, with 25–35% presenting germline pathogenic variants [20,21]. Additionally, *SMARCB1* mutations have also been described in other malignancies of the female genital tract as undifferentiated endometrial carcinoma and vulval neoplasms [11]. The morphological and molecular similarities between SCCOHT and RTs led some authors to suggest that SCCOHT should be considered malignant rhabdoid tumors of the ovary [22]. However, other authors provided molecular arguments based on DNA methylation and transcriptomic profiling that the *SMARCA4*-deficient RTs present molecular features distinct from the *SMARCB1*-deficient RTs and the *SMARCA4*-deficient SCCOHT [23,24], supporting their separate classification. Regarding the *SMARCB1*-deficient SCCOHTs, there are only three cases reported in medical literature [17] and unfortunately, no details regarding morphological or molecular features were described.

Due to the risk of developing other malignances, risk-reducing contralateral oophorectomy for SCCOHT patients is discussed, as well as for patients who carry a germline *SMARCA4* pathogenic variant mutation but who do not develop SCCOHT. Since there is a lack of evidence that surveillance can prevent death from SCCOHT, the ISC does not recommend it [1].

There are few trials concerning SCCOHT management and no consensus regarding the best treatment option. Although the approach to this malignance is based on case reports, there are many ongoing clinical trials. A multimodal approach includes primary cytoreductive surgery, radiotherapy, and chemotherapy. The choice of a regimen is extrapolated from the small cell lung carcinoma data and the most frequently used adjuvant chemotherapies are combinations based on cisplatin and etoposide [25]. The most common protocols are based on cisplatin, doxorubicin, etoposide, and cyclophosphamide (PAVEP protocol); and vinblastine, cisplatin, cyclophosphamide, bleomycin, doxorubicin, and etoposide (VPCBAE protocol) [1].

There is some published data demonstrating the benefit of the addition of high-dose chemotherapy (HDC) to standard-dose adjuvant chemotherapies. A prospective clinical trial published in 2020 enrolled patients with SCCOHT who underwent optimal cytoreductive surgery to receive PAVEP for four to six cycles. In case of a complete response, patients received HDC with stem cell transplantation (SCT), followed by pelvic radiotherapy. HDC was significantly associated with better overall survival (*p* < 0.001), and despite the increase in grades 3/4 adverse events, such as mucositis and nausea, the toxicity was manageable [26].

In a previous prospective trial published in 2007, a multicentric cohort of 27 SCCOHT patients was treated with multi-agent combination PAVEP for four to six cycles with optimal, non-conservative cytoreductive surgery. In the event of a complete response, patients received HDC with autologous SCT. The results showed a 3-year overall survival rate of 49% for all stages [27]. According to this data, the ICS suggests multimodality therapy with surgery and multi-agent chemotherapy with possible SCT an effective approach for SCCOHT.

Some clinical studies testing other treatments are ongoing, with a focus on epigenetic therapies. The *SMARCA4* deficient cancer cells display sensitivity to suppression of the enhancer of zeste homolog 2 (EZH2). In SCCOHT tumors, EZH2 inhibitors induce the expression of the *SMARCA2* [28]. A phase II single-arm trial that includes patients with SCCOHT who are treated with tazemetostat, an EZH2 inhibitor, is ongoing (NCT02601950). After treatment, early results reported that only a subset of patients with SCCOHT is likely to benefit from the use of EZH2inh.

Kinase inhibitors such as CDK4/6 inhibitors also have activity in the treatment of SCCOHT. *SMARCA4* loss causes downregulation of cyclin D1, which limits CDK4/6 kinase activity in the SCCOHT cells and leads to the susceptibility to CDK4/6 inhibitors. An article published in 2019 proved that CDK4/6 inhibitors, approved for a breast cancer subtype, could be used to treat SCCOHT [29]. However, more clinical trials are needed to improve this approach.

Although the low TMB of SCCOHT would not predict responsiveness to an immune checkpoint blockade (ICB), programmed cell death protein 1 (PD-1) inhibitors with pembrolizumab have shown a substantial and durable responses in selected patients. These patients were treated with cytotoxic chemotherapy immediately following radiation and the disease recurred after a disease-free interval of one to three years. While unexpected for a low TMB, these tumors demonstrated PD-L1 expression with strongly associated T-cell infiltration [30], which probably explains these positive findings.

## 4. Conclusions

The rarity of SCCOHT poses a challenge for the correct diagnosis and management of affected patients. In the case described in this report, the patient was diagnosed with SCCOHT with a rare *SMARCB1* somatic mutation instead of the presence of an expected *SMARCA4* alteration. This case report emphasizes the importance of performing INI1/*SMARCB1* IHC on undifferentiated ovarian tumors in young patients that show a retained *SMARCA4* expression to determine the correct diagnosis of SCCOHT.

The patient in this report received chemotherapy, but rapidly developed disease progression and died before it was possible to initiate other treatments. Multi-institutional collaboration is warranted to address the challenges of a complete characterization and the development of more efficient treatments for this rare and deadly disease.

## Supplementary Materials

The following supporting information can be downloaded at: https://www.mdpi.com/article/10.3390/curroncol29020037/s1, Supplementary File S1: Sample collection and DNA/RNA extraction; Targeted somatic panel analysis; Targeted amplicon analysis.
