# Supplementary material for: Case Report of Small Cell Carcinoma of the Ovary, Hypercalcemic Type (Ovarian Rhabdoid Tumor) with SMARCB1 Mutation: A Literature Review of a Rare and Aggressive Condition
Source: Curr Oncol. 2022 Jan 18;29(2):411–22. doi: 10.3390/curroncol29020037 (PMC8870484; doi:10.3390/curroncol29020037)
Supplement: Supplementary file 1 [file curroncol-29-00037-s001.zip › curroncol-1548127-supplementary.pdf]

SUPPLEMENTARY FILE S1

Sample collection and DNA/RNA extraction:

For germline genetic testing, whole blood was collected in BD Vacutainer® EDTA Tube (BD Biosciences, NJ, USA) and submitted to centrifugation for isolation of leukocytes. DNA was extracted from using the QIAsymphony DNA Mini Kit (Qiagen, Hilden, Germany), according to the manufacturer's instructions.

For somatic analysis, tumor specimen from formalin-fixed paraffin-embedded (FFPE) block was reviewed by a medical pathologist to assess the percentage of tumor cells and mark tumor areas. Genomic DNA was extracted from unstained slides after paraffin removal and manual dissection of selected tumor regions using the AllPrep DNA/RNA Mini Kit (Qiagen, Hilden, Germany).

For liquid biopsy analysis, 8 mL of peripheral blood was collected in PAXgene Blood ccfDNA Tube (Qiagen, Hilden, Germany) and submitted to two rounds of centrifugation at 1,600 g for 10 min within 2 hours of collection. DNA was extracted from the plasma using the MagMAX Cell-Free DNA Isolation Kit (Thermo Fisher Scientific, MA, USA), according to the manufacturer's instructions.

For all samples, DNA extractions were performed at A.C.Camargo Biobank and followed standardized procedures. DNA quality was assessed with TapeStation DNA kits (Agilent Technologies) and DNA quantity was assessed with Qubit dsDNA HS kit (Thermo Fisher Scientific, MA, USA).

Germline genetic testing:

Germline genetic testing was performed in leucocyte DNA using a custom gene panel from Sophia Genetics. This test allows to detect alterations in the coding regions and in the splicing regions (10 base pairs of the intronic regions that are adjacent to the exons) of the 112 genes (Table 1). Target regions were captured using a customize kit from Sophia Genetics and next generation sequencing was performed on Illumina MiniSeq. Resulting data was analyzed with Sophia Genetics DDM software to detect single-base point mutations, small deletions, or insertions and copy number variations (CNV). A minimum of 50 reads was obtained for all targeted bases. The classification and interpretation of the variants was performed following American College of Medical Genetics (ACMG) guidelines.

Table S1. 112 cancer predisposing genes evaluated in germline genetic testing.

ACD	*BMPR1A*	*CDKN1C*	*ERCC2*	*HNF1A*	*MLH3*	*NTHL1*	*PRSS1*	*RUNX1*	*SMARCE1*	*VHL*
AIP	*BRCA1*	*CDKN2A*	*EXT1*	*HRAS*	*MRE11*	*PALB2*	*PTCH1*	*SDHA*	*STK11*	*WRN*
AKT1	*BRCA2*	*CEBPA*	*EXT2*	*KIF1B*	*MSH2*	*PDGFRA*	*PTEN*	*SDHAF2*	*SUFU*	*WT1*
ALK	*BRIP1*	*CHEK2*	*FANCC*	*KIT*	*MSH3*	*PHOX2B*	*RAD50*	*SDHB*	*TERC*	*XPA*
APC	*BUB1B*	*CREBBP*	*FANCG*	*LZTR1*	*MSH6*	*PMS2*	*RAD51C*	*SDHC*	*TERF2IP*	*XPC*
ATM	*CASR*	*CTNNA1*	*FANCM*	*MAX*	*MUTYH*	*POLD1*	*RAD51D*	*SDHD*	*TERT*	*XRCC2*
AXIN2	*CDC73*	*DICER1*	*FH*	*MC1R*	*NBN*	*POLE*	*RB1*	*SLX4*	*TMEM127*	
BAP1	*CDH1*	*DIS3L2*	*FLCN*	*MEN1*	*NF1*	*POLH*	*RECQL4*	*SMAD4*	*TP53*	
BARD1	*CDK4*	*EGLN1*	*GALNT12*	*MET*	*NF2*	*POT1*	*RET*	*SMARCA4*	*TSC1*	
BLM	*CDKN1B*	*EPCAM*	*GATA2*	*MLH1*	*NSD1*	*PRKAR1A*	*RHBDF2*	*SMARCB1*	*TSC2*	

Targeted somatic panel analysis:

Somatic mutations in tumor DNA were investigated by targeted next generation sequencing (NGS) using the AmpliSeq Comprehensive Cancer Panel (Thermo Fisher Scientific). This panel evaluates the coding regions of 409 genes frequently mutated in tumors (table 2). Multiplex amplification was performed with 10 ng of paired tumor and leucocyte DNA using the Ion AmpliSeq Library Kit 2.0 (Thermo Fisher Scientific), and high-throughput sequencing was performed using the Ion Proton platform (Thermo Fisher Scientific), according to the manufacturer's instructions. Variant calling and

annotation were performed with Ion Torrent Variant Caller and Ion Report software (Thermo Fisher Scientific) using commercial pipelines. Variant annotation and filtration were additionally performed using VarSeq software (GoldenHelix). Variants were selected when they presented variant allele frequency >5%, coverage >100 reads, and absence of leukocyte DNA.

Table S2. 409 cancer genes evaluated in tumor genomic testing.

ABL1	*BUB1B*	*DEK*	*FLT1*	*ITGA9*	*MLL*	*PALB2*	*RALGDS*	*TCF12*
ABL2	*CARD11*	*DICER1*	*FLT3*	*ITGB2*	*MLL2*	*PARP1*	*RARA*	*TCF3*
ACVR2A	*CASC5*	*DNMT3A*	*FLT4*	*ITGB3*	*MLL3*	*PAX3*	*RB1*	*TCF7L1*
ADAMTS20	*CBL*	*DPYD*	*FN1*	*JAK1*	*MLLT10*	*PAX5*	*RECQL4*	*TCF7L2*
AFF1	*CCND1*	*DST*	*FOXL2*	*JAK2*	*MMP2*	*PAX7*	*REL*	*TCL1A*
AFF3	*CCND2*	*EGFR*	*FOXO1*	*JAK3*	*MN1*	*PAX8*	*RET*	*TET1*
AKAP9	*CCNE1*	*EML4*	*FOXO3*	*JUN*	*MPL*	*PBRM1*	*RHOH*	*TET2*
AKT1	*CD79A*	*EP300*	*FOXP1*	*KAT6A*	*MRE11A*	*PBX1*	*RNASEL*	*TFE3*
AKT2	*CD79B*	*EP400*	*FOXP4*	*KAT6B*	*MSH2*	*PDE4DIP*	*RNF2*	*TGFBR2*
AKT3	*CDC73*	*EPHA3*	*FZR1*	*KDM5C*	*MSH6*	*PDGFB*	*RNF213*	*TGM7*
ALK	*CDH1*	*EPHA7*	*G6PD*	*KDM6A*	*MTOR*	*PDGFRA*	*ROS1*	*THBS1*
APC	*CDH11*	*EPHB1*	*GATA1*	*KDR*	*MTR*	*PDGFRB*	*RPS6KA2*	*TIMP3*
AR	*CDH2*	*EPHB4*	*GATA2*	*KEAP1*	*MTRR*	*PER1*	*RRM1*	*TLR4*
ARID1A	*CDH20*	*EPHB6*	*GATA3*	*KIT*	*MUC1*	*PGAP3*	*RUNX1*	*TLX1*
ARID2	*CDH5*	*ERBB2*	*GDNF*	*KLF6*	*MUTYH*	*PHOX2B*	*RUNX1T1*	*TNFAIP3*
ARNT	*CDK12*	*ERBB3*	*GNA11*	*KRAS*	*MYB*	*PIK3C2B*	*SAMD9*	*TNFRSF14*
ASXL1	*CDK4*	*ERBB4*	*GNAQ*	*LAMP1*	*MYC*	*PIK3CA*	*SBDS*	*TNK2*
ATF1	*CDK6*	*ERCC1*	*GNAS*	*LCK*	*MYCL1*	*PIK3CB*	*SDHA*	*TOP1*
ATM	*CDK8*	*ERCC2*	*GPR124*	*LIFR*	*MYCN*	*PIK3CD*	*SDHB*	*TP53*
ATR	*CDKN2A*	*ERCC3*	*GRM8*	*LPHN3*	*MYD88*	*PIK3CG*	*SDHC*	*TPR*
ATRX	*CDKN2B*	*ERCC4*	*GUCY1A2*	*LPP*	*MYH11*	*PIK3R1*	*SDHD*	*TRIM24*
AURKA	*CDKN2C*	*ERCC5*	*HCAR1*	*LRP1B*	*MYH9*	*PIK3R2*	*SEPT9*	*TRIM33*
AURKB	*CEBPA*	*ERG*	*HIF1A*	*LTF*	*NBN*	*PIM1*	*SETD2*	*TRIP11*
AURKC	*CHEK1*	*ESR1*	*HLF*	*LTK*	*NCOA1*	*PKHD1*	*SF3B1*	*TRRAP*
AXL	*CHEK2*	*ETS1*	*HNF1A*	*MAF*	*NCOA2*	*PLAG1*	*SGK1*	*TSC1*
BAI3	*CIC*	*ETV1*	*HOOK3*	*MAFB*	*NCOA4*	*PLCG1*	*SH2D1A*	*TSC2*
BAP1	*CKS1B*	*ETV4*	*HRAS*	*MAGEA1*	*NF1*	*PLEKHG5*	*SMAD2*	*TSHR*
BCL10	*CMPK1*	*EXT1*	*HSP90AA1*	*MAGI1*	*NF2*	*PML*	*SMAD4*	*UBR5*
BCL11A	*COL1A1*	*EXT2*	*HSP90AB1*	*MALT1*	*NFE2L2*	*PMS1*	*SMARCA4*	*UGT1A1*
BCL11B	*CRBN*	*EZH2*	*ICK*	*MAML2*	*NFKB1*	*PMS2*	*SMARCB1*	*USP9X*
BCL2	*CREB1*	*FAM123B*	*IDH1*	*MAP2K1*	*NFKB2*	*POT1*	*SMO*	*VHL*
BCL2L1	*CREBBP*	*FANCA*	*IDH2*	*MAP2K2*	*NIN*	*POU5F1*	*SMUG1*	*WAS*
BCL2L2	*CRKL*	*FANCC*	*IGF1R*	*MAP2K4*	*NKX2-1*	*PPARG*	*SOCS1*	*WHSC1*
BCL3	*CRTC1*	*FANCD2*	*IGF2*	*MAP3K7*	*NLRP1*	*PPP2R1A*	*SOX11*	*WRN*
BCL6	*CSF1R*	*FANCF*	*IGF2R*	*MAPK1*	*NOTCH1*	*PRDM1*	*SOX2*	*WT1*
BCL9	*CSMD3*	*FANCG*	*IKBKB*	*MAPK8*	*NOTCH2*	*PRKAR1A*	*SRC*	*XPA*
BCR	*CTNNA1*	*FANCJ*	*IKBKE*	*MARK1*	*NOTCH4*	*PRKDC*	*SSX1*	*XPC*
BIRC2	*CTNNB1*	*FAS*	*IKZF1*	*MARK4*	*NPM1*	*PSIP1*	*STK11*	*XPO1*
BIRC3	*CYLD*	*FBXW7*	*IL2*	*MBD1*	*NRAS*	*PTCH1*	*STK36*	*XRCC2*
BIRC5	*CYP2C19*	*FGFR1*	*IL21R*	*MCL1*	*NSD1*	*PTEN*	*SUFU*	*ZNF384*
BLM	*CYP2D6*	*FGFR2*	*IL6ST*	*MDM2*	*NTRK1*	*PTGS2*	*SYK*	*ZNF521*
BLNK	*DAXX*	*FGFR3*	*IL7R*	*MDM4*	*NTRK3*	*PTPN11*	*SYNE1*	
BMPR1A	*DCC*	*FGFR4*	*ING4*	*MEN1*	*NUMA1*	*PTPRD*	*TAF1*	
BRAF	*DDB2*	*FH*	*IRF4*	*MET*	*NUP214*	*PTPRT*	*TAF1L*	
BRD3	*DDIT3*	*FLCN*	*IRS2*	*MITF*	*NUP98*	*RAD50*	*TAL1*	
BTK	*DDR2*	*FLI1*	*ITGA10*	*MLH1*	*PAK3*	*RAF1*	*TBX22*	

Targeted amplicon analysis:

For liquid biopsy analyses, the somatic mutations identified in the tumor (*SMARCB1* and *PTEN*) were investigated using specific amplicons. PCR products were amplified using the Qiagen Multiplex PCR Kit (Qiagen) and libraries were prepared with the Ion Plus Fragment Library Kit (Thermo Fisher Scientific). High-throughput sequencing was performed using the Ion Proton platform (Thermo Fisher Scientific), according to the manufacturer's instructions. Visual inspection of base calling and absolute base frequencies annotation were performed using Integrative Genome Viewer (IGV) (Robinson et al., 2011). The mutation was considered present if the variant frequency was >0.5%; a minimum coverage depth of 20,000X was considered for negative results.
